# Largest Cretaceous lizard track assemblage, new morphotypes and longest trackways comprise diverse components of an exceptional Korean Konservat-Lagerstätten ichnofauna

**DOI:** 10.1038/s41598-019-49442-0

**Published:** 2019-09-16

**Authors:** Kyung Soo Kim, Jong Deock Lim, Martin G. Lockley, Dong Hee Kim, Laura Piñuela, Jae Sang Yoo

**Affiliations:** 1 Department of Science Education, 3 Jinnyangho-ro 369beon-gil, Jinju-si, Gyeongnam, 52673 Korea; 2 Cultural Heritage Administration, Government Complex-Daejeon, 189, Cheongsa-ro, Seo-gu, Daejon, 35208 Korea; 3 Dinosaur Trackers Research Group, University of Colorado Denver, P.O. Box 173364, Denver, CO 80217-3364 USA; 4 National Science Museum, 481 Daedeok-daero, Yuseong-gu, Daejeon, 34143 Korea; 5 Museo del Jurásico de Asturias (MUJA), Rasa de San Telmo, s/n, 33328 Colunga, Asturias, Spain

**Keywords:** Palaeontology, Palaeontology

## Abstract

A newly discovered assemblage of lizard tracks from the Lower Cretaceous Jinju Formation (Sindong Group, Gyeongsang Basin) Korea is the largest yet reported from the Cretaceous. It consists of at least 95 tracks comprising five trackways, including a meter-long trackway (T1) with 50 footprints assigned to the new ichnotaxon *Neosauroides innovatus* ichnosp. nov. Two other trackways (T2 and T3) are designated *N. innovatus* paratypes characterized by strong heteropody, relatively wide trackways and small narrow manus tracks. These morphological characteristics distinguish *Neosauroides innovatus* from the previously reported lizard trackways *Sauripes hadongensis* from the Hasandong Formation and *Neosauroides koreaensis* from the Haman Formation, both also from the Gyeongsang Basin. These three lizard track assemblages from the Korean Cretaceous constitute the entire global lizard track record for this period. The Mesozoic record of lizard tracksites is more localized than the lizard body fossil record. This limited distribution suggests bias in the track record and the fossil record more generally. However, due to deposition of fine-grained substrates, suitable for high definition track registration, the Jinju Formation is increasingly well known as an ichnological window on small tetrapod activity and based on diversity, abundance and high-quality preservation, is regarded as an exceptional Konservat-Lagerstätten.

## Introduction

**Figure 1:**
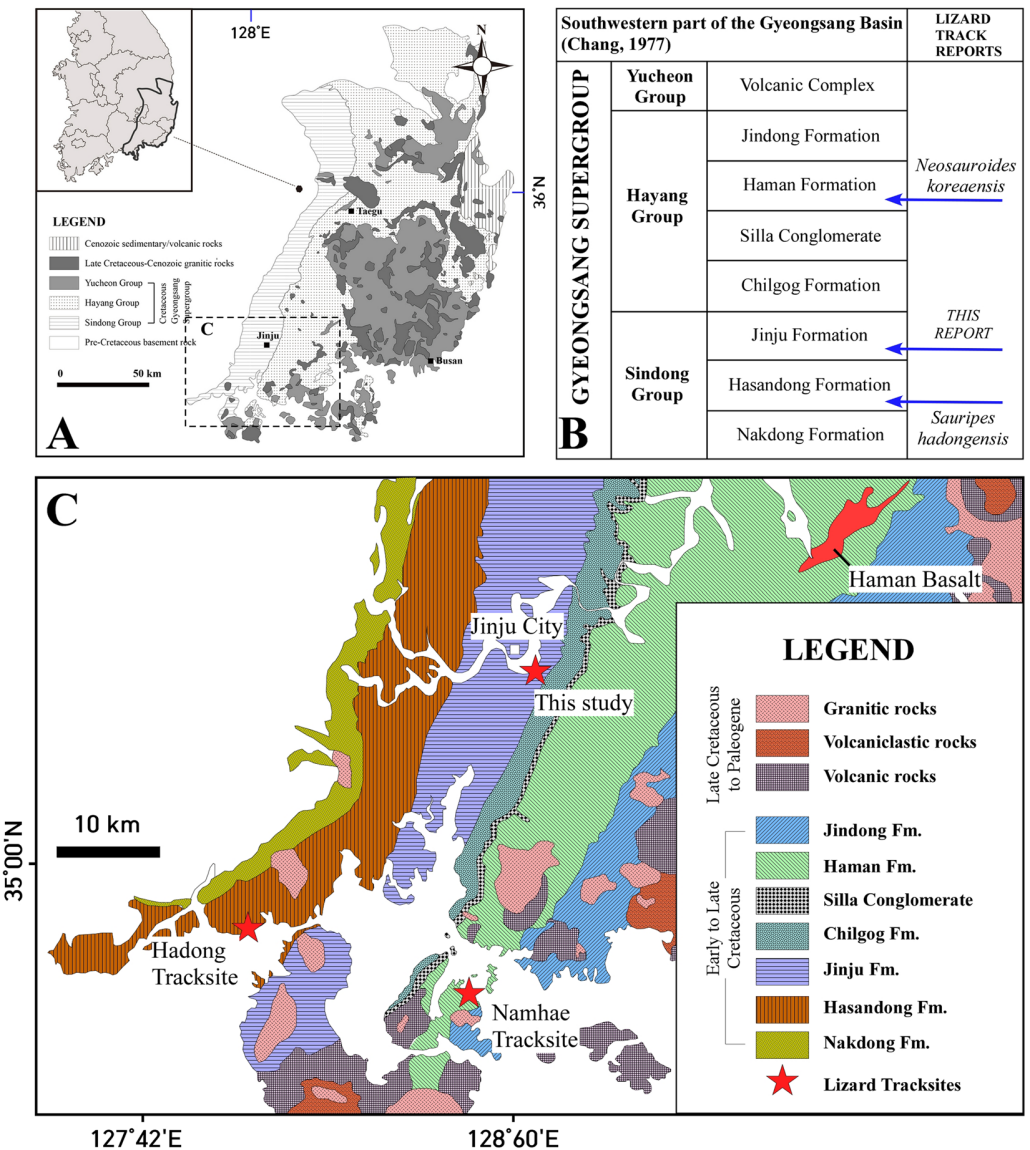
The geological map (**A**), stratigraphy (**B**), and three lizard tracksites (**C**) of the Gyeongsang Basin. Maps made by K-S K and M G L in Adobe photoshop (version CS6 88) and Canvas X (version, 2017 Build 160, http://www.canvasgfx.com/).

No fossil footprints of lizards had been reported from the Cretaceous of Asia, indeed from the entire global Cretaceous, prior to 2017. This situation changed with the report of a single trackway, comprising eight tracks, from the Haman Formation of the Hayang Group (Fig. 1), from Changseon Island, Korea^1^. The trackway was named as *Neosauroides koreaensis*, and shown to be distinct from various ichnospecies in the ichnogenus *Rhynchosauroides* which are common in the Late Triassic, as noted below. Again in 2017, additional lizard tracks were reported from the Hasandong Formation^2^, which forms part of the Sindong Group, which underlies the Hayang Group. These tracks had previously been reported in an obscure survey report in Korean and interpreted as amphibian tracks^3^. In the 2017 update, the tracks were characterized as the oldest known “modern lizard trackways”^2^ comprising an assemblage of 29 tracks, of which 25 were interpreted as pes tracks and four as manus tracks. In a third study^4^ the Hasandong Formation tracks were shown to comprise four trackways, with 10, 10, 4 and 4 tracks respectively (A1-A10, B1-B10, C1-C4 and D1-D4,) and an isolated partial track (E1). The tracks were named *Sauripes hadongensis*, interpreting tracks A10 and B1, B3 and B4 as manus tracks, and the remaining tracks as pes impressions. Due to the lack of manus tracks, and the reduced outward rotation of the pes tracks, the tracks in the A and B trackways were interpreted as evidence of running, an inference that is discussed below.

Here we report another lizard track assemblage from the Jinju Formation, overlying the Hasandong Formation, in the Jinju City area (Fig. 1). The Jinju Formation is the youngest unit of the Sindong Group. Thus, the stratigraphic position of the Jinju discovery is intermediate between the older levels from which the Hasandong specimen originated, and the younger levels from which the Haman specimen was recovered. The Jinju discovery, detailed here, reveals 95 tracks comprising the trackways of five different trackmakers (SI), and is the largest lizard track assemblage currently known from the Cretaceous or the Jurassic. Current evidence indicates that since 2017 the known sample of Cretaceous lizard footprints has increased from zero to a total of >130, representing 10 different trackways, and is based entirely on the Korean track record from three different formations.

## Geological Setting

The lizard tracks from the Jinju Formation described in this study were discovered from the 2^nd^ Excavation Project (“Excavation 2”) at the construction site of Jinju Innovation City. The 2^nd^ excavation tracksite has also yielded vertebrate tracks such as the smallest raptor (*Dromaeosauriformipes*)^5^, theropod, small crocodylomorph, other tetrapod tracks and invertebrate trace fossil, *Protovirgularia* ichnosp. The stratigraphic level of the 2^nd^ excavation tracksite (Fig. S1) is lower than the 3^rd^ and 4^th^ excavation sites^6–9^ and higher than the 1st excavation site.

**Figure 2:**
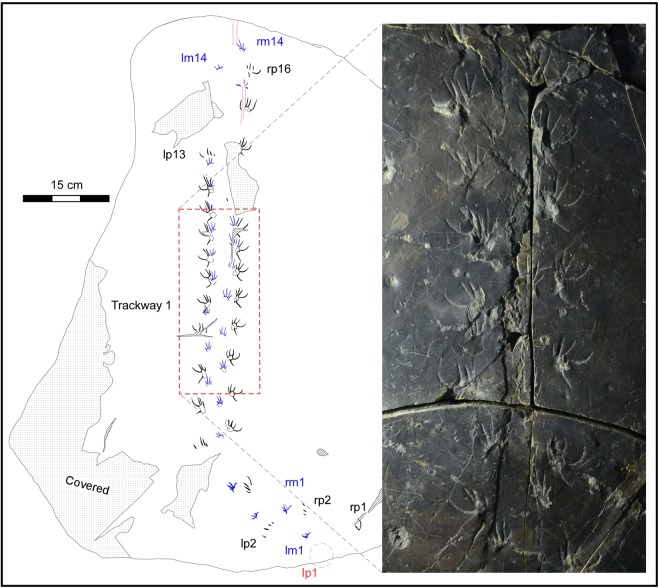
Map of trackway T1 (left), the holotype of *Neosauroidies innovatus* ichnosp. nov., with photograph (right) of middle portion of trackway. See Supplementary Information (Figs SI 2, 3) and Table SI 1, for additional illustrations, photographs and maps showing all trackways (T1-T5) and morphometric parameters measured. Inferred position of left pes 1 (lp1) shown with red label. Drawing created with Canvas X (version, 2017 Build 160, http://www.canvasgfx.com/). Photograph by K-S Kim.

**Figure 3:**
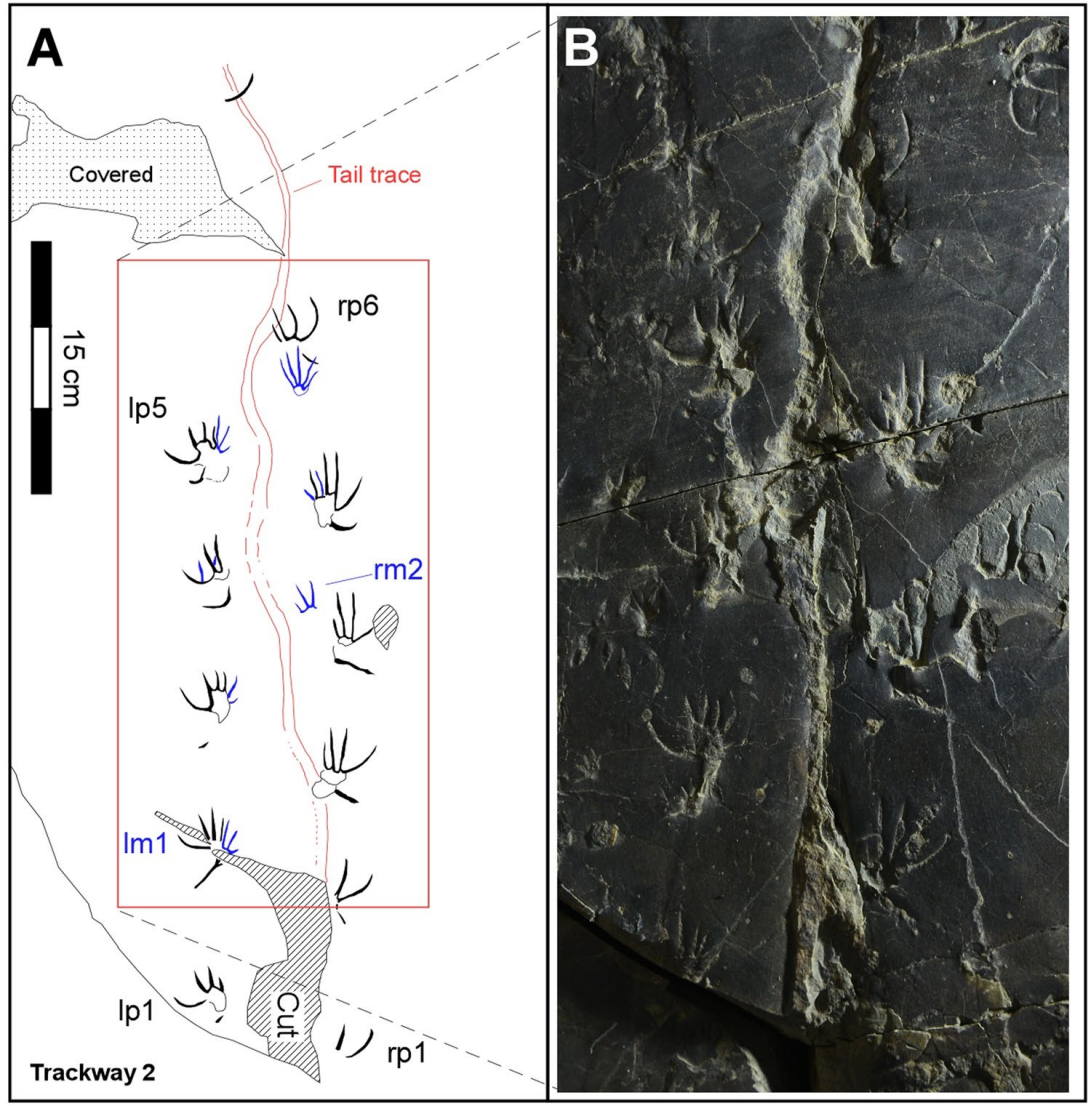
Map (left) and photo (right) of trackway T2 showing sinuous tail traces. See text for details and compare with Fig. SI 2 for position of trackway T2 relative to other trackways (T1, T3-T5). Drawing created with Canvas X (version, 2017 Build 160, http://www.canvasgfx.com/). Photograph by K-S Kim.

**Figure 4:**
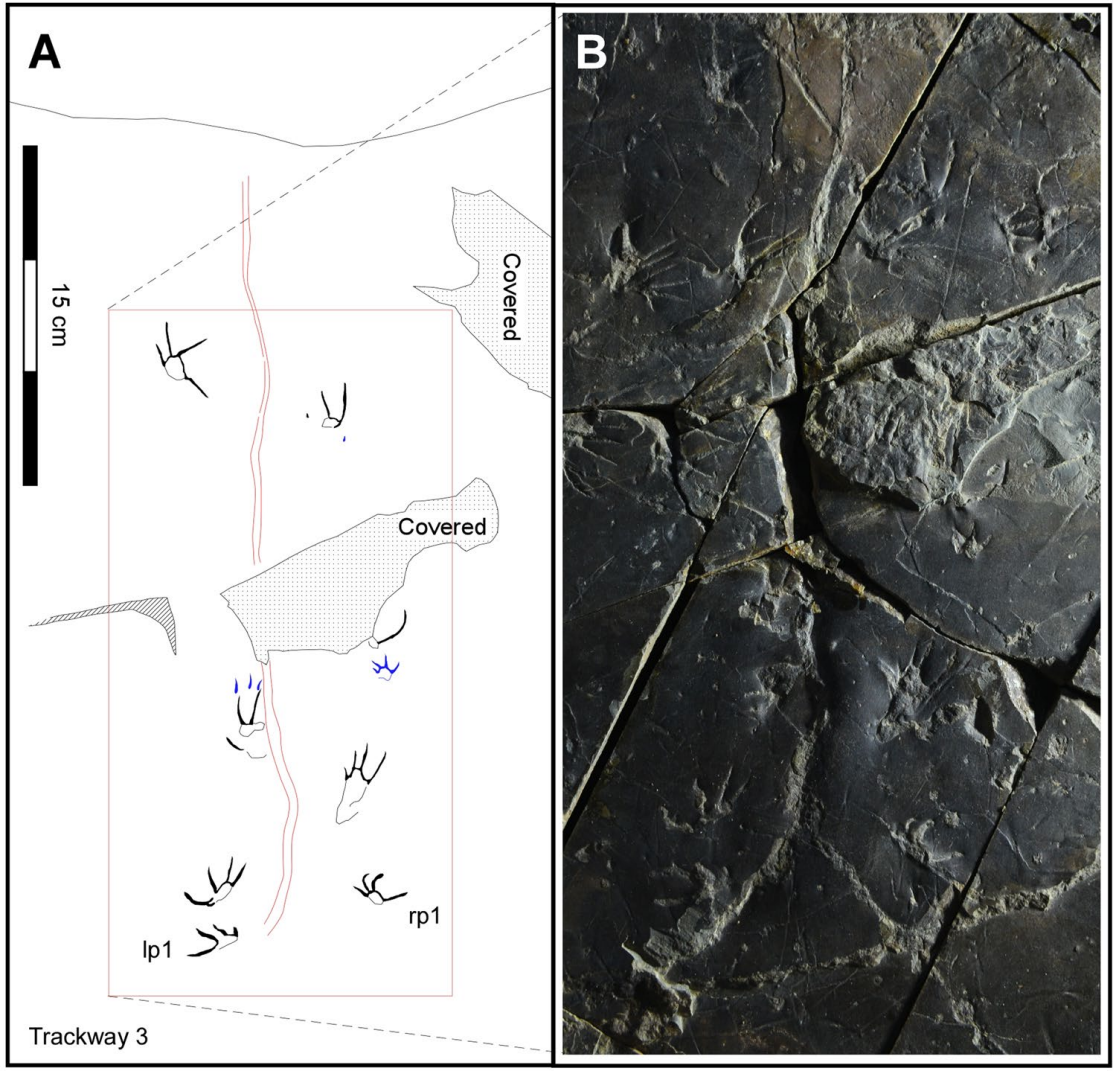
Map (left) and photograph (right) of trackway T3. See text for details and compare with Fig. SI 2 for position of trackway T3 relative to other trackways (T1, T2, T 4 and T5). Drawing created with Canvas X (version, 2017 Build 160, http://www.canvasgfx.com/). Photograph by K-S Kim.

**Figure 5:**
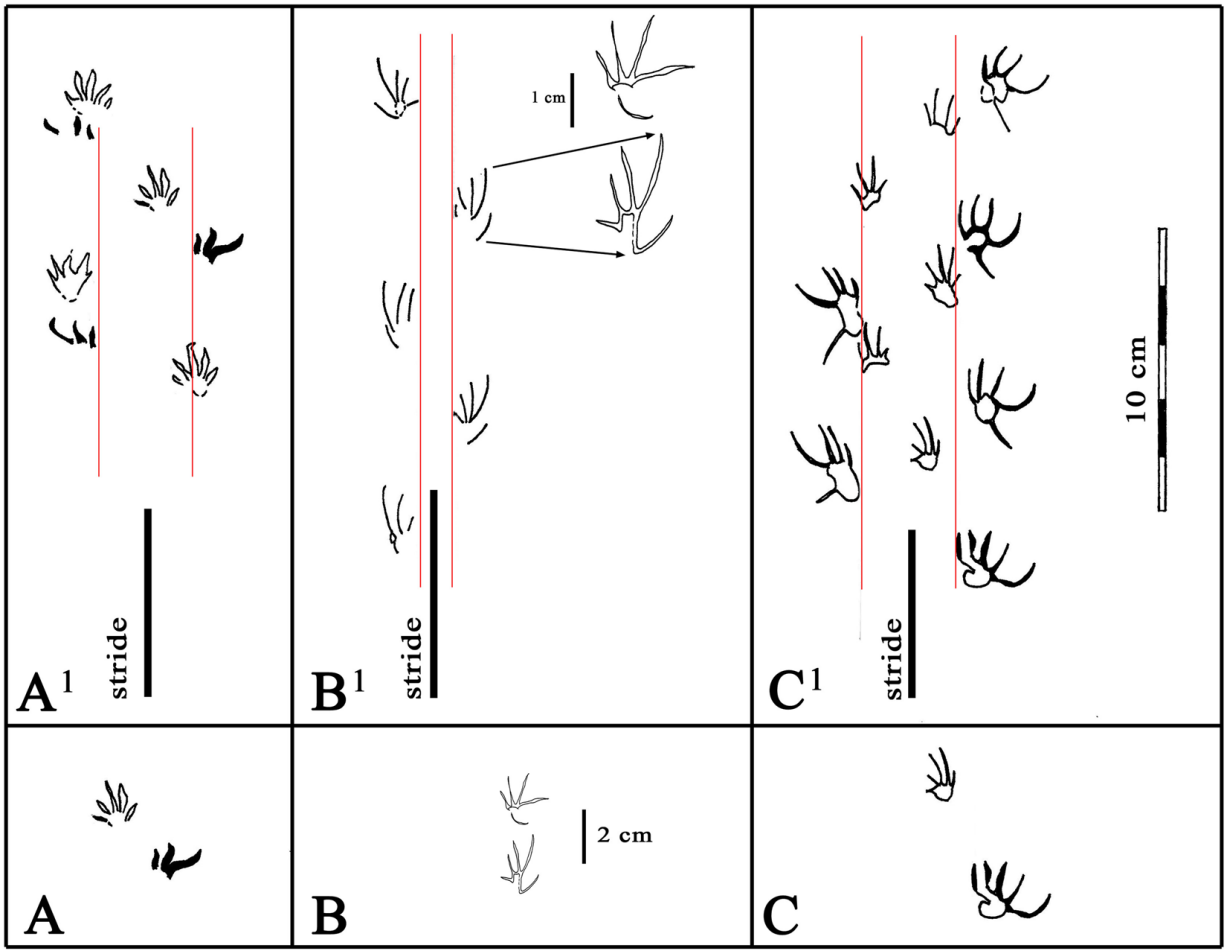
Comparison of *Neosauroides koreaensis* (A-A^1^)^1^ *Sauripes hadongensis* (B-B^1^)^4^ and *Neosauroides innovatus* ichnosp. nov. (C-C^1^), this paper. (**A**–**C**) show right manus pes sets at same scale, based on published record. A^1^-C^1^ show corresponding trackways, at same scale, with inner pes trackway widths marked with red lines, and stride lengths marked with heavy black lines. Details of pes and purported manus of *S. hadongensis* shown at double trackway scale in frame B^1^. Note that we dispute the interpretation of the S. *hadongensis* manus: see text for details. Drawing created with CorelDRAW X8 Graphic, version 18.1.0.661, https://www.coreldraw.com.

The trackways described here originate from a series of grey shales/mudstones, siltstones, alternating fine-grained sandstones and mudstones, and fine-grained sandstones of the Lower Cretaceous Jinju Formation which makes up part of the Sindong Group within the Gyeongsang Supergroup from near Jinju City, South Korea (Fig. 1B and SI 1). These deposits represent a lake basin paleoenvironment that was extremely rich in track-bearing units, with eight track-bearing levels previously reported from a 2.6 meter section that yielded avian (bird) and non-avian, theropod, ornithopod and pterosaur tracks from only one of four excavation sites, in a thick sequence of lacustrine basin sediments^7^. The lizard trackways described here (Figs 2–5, and SI 2–5) from the 2^nd^ excavation site represent a separate assemblage from those found at the 3^rd^ and 4^th^ excavation sites and detailed in two reports, in Korean^8,9^ as well as in mainstream literature reports on the first mammal tracks from the Cretaceous of Asia^6^ and an important assemblage of theropod tracks^7^. The Jinju Formation is considered Aptian in age^10^.

## Material and Methods

The large track-bearing slab (Fig. SI 1), designated as specimen number CUE JI-2E Li001 in the Chinju National University of Education (CUE) collections is already on display at the *Jinju Pterosaur Footprint Museum*. The slab reveals a surface with maximum length-width dimensions of ~100 cm and ~80 cm, broken by a few narrow fractures. The surface is very flat except in a few places where it is broken and layers of the overlying bed adhere. The trackways are preserved as shallow positive impressions (concave epireliefs), but we also made a silicon mold (convex hyporeliefs) so as to create a replica in polyurethane resin. The tracks are mostly very shallow (~1.0 mm) where not filled, but well-preserved and complete, mostly revealing clear trackway trends, and impressions of all five digits. In addition to taking photographs (Fig. SI 1), we used clear acetate film to trace the tracks with fine point pens (Fig. 2). These tracings form the basis of the line-drawing maps presented here (Figs 2–5 and SI 2–5). Through visual inspection of the original specimen, the polyurethane replica and the tracings we identified five different trackways with footprints varying in maximum dimensions from ~47.70 mm–13.83 mm. We designated the five trackways as T1-T5 in decreasing order of size and length of trackway, and in most cases were able to distinguish between the smaller manus and larger pes tracks and trace and represent them graphically with different colors (Figs 2–3 and SI 2–5). In some cases we could also trace faint tail traces. Trackways T1-T3 are described in this order in the following section, with trackways 4-5 described in the Supplementary Information (SI). Summary and detailed measurements for all tracks are provided in Tables SI 1-2. The tracks were too shallow, in some cases with no relief, to obtain useful 3D images, superior to 2D photographs and tracings, using available photogrammetric techniques (SI).

### Description of tracks and trackways

#### Trackway T1

Trackway T1 is the longest trackway consisting of 26 pes and 24 manus tracks (shown in black and blue respectively in Fig. 2 and SI 2, 3): total 50 tracks. Trackway T1 is also designated as the holotype of *Neosauroides innovatus*, ichnosp. nov., described below. Although the trackway is registered across the whole track-bearing surface sub parallel to the long axis of the slab (Fig. 2 and SI 2, 3), it is difficult to assign each manus and pes a sequential number (e.g., T1rp1, rm1, rp2 etc.,) because of the few places where tracks are missing due to damage or poor preservation. The first formed (more-proximal) seven tracks, especially those of the pes are less complete than those in the middle section of the trackway, and appear to indicate longer steps. This middle portion of trackway T1 indicates a 70° turn to the right, relative to the proximal portion. The later-formed, distal portion of the trackway, consisting of the last five tracks, is also less well-preserved, partly due to damage to the track surface, but a very faint tail trace is discernible in this portion. The proximal manus to manus steps are longer than those in mid trackway, and represent accelerated locomotion. The middle portion of the trackway consisting of the remaining 37 tracks is straight and clearly indicates a shortened step was registered. Thus, the middle part of trackway T1 indicates a deceleration of speed: see SI.

Pes tracks are clearly pentadactyl and ectaxonic with digit IV the longest and digit I the shortest (IV > II = III > V > I). The digits register in a highly splayed, sub radial pattern with maximum track dimension about 38.92 mm (Fig. 4C). Mean divarication angle between digit traces IV and V (~77°) is the greatest followed by that between IV and III (~37°), with mean digit divarication angles between III and II and II and I, about 9° and 13°, respectively. The outer digit (V) trace is slightly curved (concave inward towards digit IV trace) but registered postero-laterally (outwardly) at an angle of 122–164° (mean 146°) to the trackway midline. The longest digit IV is strongly curved (concave inward towards digit III) and generally registered antero-laterally at an angle of ~ −12–83° (mean 62°) to the trackway midline. Digit traces III, II and I are oriented progressive more anteriorly, but still laterally, at angle averages of about 25°, 14° and 2° to the mid line. The outer trackway width for the pes is ~88–93 mm, inner trackway width ~29–34 mm: i.e., inner width is slightly greater than maximum pes diameter (Table SI 1).

Manus tracks pentadactyl and ectaxonic with digit traces IV the longest, digit III slightly shorter and II and V subequal in length (IV > III > II = V > I). Digit traces II, III and IV typically sub-parallel to slightly splayed with digit III sub-parallel to trackway axis. Manus elongate about one and a half times as long (~17 mm) as wide (~13 mm) and situated nearer the trackway mid line than the pes. The outer trackway width for the manus is up to ~57 mm and inner trackway width ~12–24 mm.

Pes step (pace) variable between ~48 and ~111 mm, with pace angulation about 64°. Stride similar to pace: i.e., between ~36 and ~181 mm. Manus step (pace) shorter than pes ~34- ~94 mm with pace angulation ~42–207°. Stride up to ~154 mm in longer proximal part of trackway.

#### Trackway T2

Trackway T2 (Fig. 3) consists of 19 complete and partial tracks including 12 pes and 7 manus tracks. We designate trackway T2 as a paratype of *N. innovatus*, ichnosp. nov. The track sizes, and configuration as well as the trackway width and step are all very similar to the pattern seen in trackway T1 (SI Table 1-2). The main difference is that trackway T2 shows a distinct sinuous, midline tail trace ~5 mm wide with a wavelength of ~100 mm and an amplitude of ~30–35 mm. It was also possible to number 11 of 12 pes tracks sequentially from right pes 1 (rp1) to rp6 (Fig. 3).

#### Trackway T3

Trackway T3 (Fig. 4) consists of 10 complete and partial tracks including 8 pes and 2 manus tracks. We designate trackway T3 as a paratype of *N. innovatus* ichnosp. nov. The track sizes, and configuration as well as the trackway width and step are all very similar to the pattern seen in trackway T1 (SI Table 1-2). The main difference is that trackway T3 shows a distinct sinuous, midline tail trace ~5 mm wide with a wavelength of ~100 mm and an amplitude of ~30–35 mm, as in trackway T2. Both pes and manus tracks are less-well-preserved than in trackways T1 and T2. For example, most pes tracks show only four digit traces, with the trace of pes digit V consistently missing. Both manus tracks are very incomplete. The incomplete preservation is due in part to damage to the track-bearing surface and the crossing of trackway T3 with trackway T1.

### Comparing the three known Cretaceous lizard trackways

As shown in Fig. 5, the trackway configurations of *Neosauroides koreaensis* (Fig. 5A,A^1^) from the Haman Formationand *Sauripes hadongensis* (Fig. 5B,B^1^) from the Hasandong Formation^4^ have been mapped to scale thus allowing comparison with tracks from the Jinju Formation. The type and only trackway of *Neosauroides koreaensis* is based on a wide trackway with well-preserved, mesaxonic manus tracks, but incomplete pes tracks which show only traces of digits II, III and IV. By contrast the type trackway of *Sauripes hadongensis* is based on a smaller, narrower trackway with only pes tracks well preserved, which prompted these authors to infer that the trackway provided evidence of running. Comparison of the Jinju trackway with these afore-named Haman and Hasandong ichnotaxa is imperative in order to determine if there are close morphological and /or ichnotaxonomic relationships. It is also important to note that the Hasandong and Jinju trackways are preserved as natural impressions (concave epireliefs) whereas the Haman Formation trackway is preserved as natural casts (convex hyporeliefs). Differences in preservation affect track morphology and potentially influence how systematic descriptions are presented.

We have concerns that the holotype of *Sauripes hadongensis* was described as “manus and pes prints on a mudstone slab (70 × 30 cm) KIGAM VP 201501”^4^ and that the illustrated pes track (A6) is from a different trackway from the illustrated manus (B1), which we consider misidentified. This is an unusual and questionable way to designate a holotype (see Systematic Discussion). In our opinion, there should be a clear distinction between a holotype slab and a chosen holotype trackway on such a slab, especially where more than one trackway was registered.

Regardless of the differences between the assemblage size, methodology, interpretation and preservation put forward in studies of the Haman, Jinju and Hasandong lizard tracks, the Jinju assemblage is the largest and includes three trackways (T1-T3) with unambiguous pes and manus tracks, which show strong heteropody. This strong heteropody clearly makes the Jinju trackway (*Neosauroides innovatus* ichnosp. nov.) quite different from *Sauripes hadongensis*. In addition there are other marked differences: i) the pes tracks of Jinju trackmakers T1-T3 are much larger and more widely splayed, ii) the manus is much smaller than the pes and less splayed (more elongate), and iii) the trackway is much wider, indicating a more sprawling gait.

Having established the differences between *Sauripes hadongensis* and *Neosauroides innovatus* ichnosp. nov., further elaborated in the Systematic Discussion, we must consider the differences between Jinju Formation *N. innovatus* ichnosp. nov. and *Neosauroides koreaensis* from the Haman Formation. In both morphotypes the manus is either quite symmetrical or slightly asymmetrical, exhibiting mesaxony with digit III the longest in *N. koreaensis* but exhibiting slight ectaxony with digit IV slightly longer than III in *N. innovatus*. Both have wide trackways. However, the manus placement is much closer to the midline in *Neosauroides innovatus* ichnosp. nov. than in *N. koreaensis*, thus giving large pace angulation values. It is difficult to compare the size of *Neosauroides innovatus* ichnosp. nov. pes tracks with those of *N*. *koreaensis* because the latter are incomplete, although apparently smaller.

**Systematic ichnotaxonomy** Class Reptilia

Infraclass Lepidosauromorpha Benton 1983

Ichnogenus *Neosauroides* Kim, Lockley, Lim, Pinuela, Xing and Moon 2017

Type ichnospecies. *Neosauroides koreaensis* Kim, Lockley, Lim, Pinuela, Xing and Moon 2017

### Neosauroides innovatus ichnosp. nov

#### Diagnosis

lacertilian trackway with strong heteropody: large splayed pentadactyl ectaxonic pes and small elongate, slightly ectaxonic, pentadactyl manus, registered close to trackway midline.

#### Differential diagnosis

*Neosauroides innovatus* ichnosp. nov., differs from con-ichnogeneric *N. koreaensis* in exhibiting strong heteropody and a more elongate manus registered much closer to the trackway midline. Pes digit traces I and V are also known in *N. innovatus* ichnosp. nov., but not in *N. koreaenisis*. *N. innovatus* differs markedly from *Sauripes hadongensis* which has a very narrow pes-dominated trackway with narrow pes tracks and incompletely known manus, lacking adequate description.

#### Description

based on a sample of three relatively wide, lacertilian trackways with strong internal pes trackway widths and less pronounced internal manus trackway widths. Heteropody pronounced. Pes large, splayed, pentadactyl, ectaxonic with digits I–III relatively straight, little divaricated, increasing in length (I < II = III < IV) and directed anteriorly to slightly anterio-laterally. Pes digit IV longest, strongly curved (convex posterior-laterally), widely divaricated from traces of digits III and V, and oriented antero-laterally to laterally. Pes digit V relatively long (IV > V = III > II > I), postero-laterally oriented and generally straighter (less posteriorly convex) than digit IV trace.

Manus elongate and more or less anteriorly directed (digit III trace ~parallel to trackway midline), with digit traces only slightly divaricated and ectaxonic: i.e., with digit IV longest (IV > III > II > V > I): see SI Tables 1-2 for measurements.

**Type horizon and locality**. Jinju Formation, Jinju Innovation City

**Age**. Early Cretaceous

**Etymology**. Ichnospecies ‘*innovatus’* named in reference to Jinju Innovation City

### Systematic Discussion

The description of the holotype slab of *Sauripes hadongensis*^4^ is problematic, creating further problems of ichnotaxonomic comparison. This slab shows multiple trackways (A–D and isolated track E). Based on the similar size and shape of purported manus track, B1^4^, and the position of the tracks on their map, we consider that it is almost certainly a pes track, moreover from a different trackway than the designated holotype pes. We suggest that this interpretation is demonstrable from simple inspection of their trackway map: i.e., the purported holotype manus track (B1) was registered exactly in the proximal position (‘prior to’ in registration sequence), to the regular right pes stride sequence B6-B8-B10 described and measured for *S. hadongensis*^4^. Arguably, it might be permissible, though questionable, to combine morphological information from two tracks, from different trackways in selecting diagnostic manus and pes in order to designate the holotype of a new ichnogenus, in this case *Sauripes*. However, it is a more serious error to mistake a pes for a manus and thus base a description on two pes tracks, one of which is incorrectly described as a diagnostic manus! This renders the ichnotaxonomic description of *S. hadongensis* deficient and thus of reduced value in comparative analysis. We consider it a potential *nomen dubium*. However, the specimen (KIGAM VP 201501) is available as well as the published map and we consider that a revised description might be salvaged using a verified manus, although we note that according to the track map none of the few preserved manus tracks registered more than three digit traces.

Based on manus tracks of *Neosauroides koreaensis* and the Jinju tracks described here, manus tracks are generally smaller and more symmetrical than in the purported manus of *S. hadongensis*^4^, here reinterpreted as a pes. This compromises comparisons with the Jinju holotype, trackway T1, which has a pes about twice and as long and wide as the manus (high heteropody). Pending revision the heteropody of *S. hadongensis* cannot be determined with any confidence.

A logical inference that could arise from accepting the original interpretation of *S. hadongensis* as a large-manus form, with low heteropody (larger anterior feet) is that it implies a front-heavy anterior emphasis biped. This is apparently inconsistent in comparison with the Jinju morphotype with its posterior emphasis (small anterior feet) that clearly progressed quadrupedally. The difference in stride length (79.18 mm) between the purported runner and Jinju trackway (up to 80 mm) is negligible, and raises questions regarding interpretation of gait and locomotion. To infer that the *S. hadongensis* trackmaker was a runner^4^ requires that the we take into account the fact that it was a smaller animal with a greater foot length-stride ratio and narrower trackway (Table SI 1).

Although ‘lizard’ or lepidosaur tracks, mostly assigned to ichnogenus *Rhynchosauroides* isp., are common in the Late Triassic^11,12^ they are comparatively rare in the Jurassic and Cretaceous. For example, we are aware of only one report of a trackway from the Jurassic^13^ and a few isolated tracks^14^ also attributed to *Rhynchosauroides* isp. To date the only known Cretaceous reports are from Korea and includes *Neosauroides koreaensis* from the Haman Formation (Fig. 1), *Sauripes hadongensis*^4^ from the Hasandong Formation, and *Neosauroides innovatus* ichnosp. nov. described here. The pes of *N. koreaensis* is incompletely known, but the trackway configuration and manus-pes differentiation are unequivocal. In contrast the manus of *S. hadongensis* is incomplete, and in our evaluation, misidentified in the type material, and therefore compromises a clear understanding of the trackway configuration. However, the manus and pes tracks of *N. innovatus* are complete and their positions in three trackways, especial the holotype trackway T1 are unequivocal. This make *N. innovatus* ichnosp. nov. the most important of the three named Korean trackway morhotypes, not just because of the size of the sample and the length of trackways, but because the individual tracks on which the ichnotaxonomy is based are complete. As discussed below and in the Supplementary Information this completeness is due to ideal, or near-ideal preservational conditions for small trackmakers.

Based on footprint length, *N. innovatus* ichnosp. nov. is about twice the size of *N. koreaensis*. *Sauripes hadongensis*^4^ is similar in size to *N. koreaensis*, but the manus appears under-represented in the sample and is poorly known and questionably-described^4^. So we do not know whether it has a mesaxonic or ectaxonic manus. The *S. hadongensis* maps suggests the manus tracks registered inside the pes at least in one trackway (B), but the trackway was also described as a pes dominated trackway, evidence inferred to indicate running^4^. However, as shown here in a comparative illustration (Fig. 5), the stride is the same as in the larger *N. koreaensis* trackway, which supposedly indicates “relatively slow speeds” and “sprawling limb posture” and increased chance of registering manus tracks^4^. As discussed below, the interpretation of the *S. hadongensis* trackmaker as a runner may be supported by the relatively narrow trackway and correspondingly high pace angulation values (SI Table 1). However, in the current literature trackway-derived speed estimates are relative and qualitative (e.g., walk, quick, very quick)^15^ as it is not possible to attach absolute or numerical values. (SI).

The heteropody of *Neosauroides koreaensis* is difficult to determine because the pes did not register fully. However, the hetropody of *N. innovatus* ichnosp. nov. is pronounced, but difficult to compare with *S*. *hadongensis* due to several problems in interpretating the manus tracks. The present study shows the potential of the Korean track record to yield more lizard trackways to facilitate our understand of the morphological diversity of trackmakers and the variability in their gaits. Based on present evidence the three named ichnospecies appear morphologically distinct from one another. This is perhaps not surprising given that they originate from three different formations. As noted below, substrate consistency and preservational factors may have played an important role in how the foot morphology of these small trackmakers was registered and preserved. *N. koreaensis* was preserved as a natural cast (convex hyporelief) whereas the other two were preserved as natural impressions (concave epireliefs).

### The fossil record of lizard tracks

It is remarkable that the global track record of Cretaceous lizards is limited to only three tracksites, all in Korea. In order of reporting, the record has yielded one *N. koreaensis* trackway^1^, four *S. hadongensis* trackways (A–D), of which two (C and D) are very incomplete^4^, and five lizard trackways of which three (T1-T3) are assigned to *N. innovatus* ichnosp. nov. and two others (T4- T5) are different but unnamed (this study). Thus, we have a sample of 10 trackways of variable completeness and quality from three different formations, and representing three different ichnospecies in two ichnogenera, one of which (*Sauripes*) is here considered in need of ichnotaxonomic revision.

Such an apparent concentration of lizard tracks in a small area could suggest a paleobiological explanation: e.g., that during the Early Cretaceous lizards were more common in the region than elsewhere. Such speculation is weak given that lizard body fossils are common in many regions, and are even diverse in some parts of Asia, e.g. Mongolia^16–18^. The alternative explanation, that facies and substrate conditions were suitable for the preferential preservation of small tetrapod tracks, is more compelling and supported by the steady increase in reports of abundant tracks attributable to small tetrapods, notably small birds, small pterosaurs, mammals and frogs^1,5–9,19–21^. In fact, on the basis of this evidence we argue that the Jinju Formation is a classic example of a Konservat-Lagerstätten defined as a deposit in which body fossil and/or trace fossils show exceptionally good preservation (see SI)^22–26^. Abundant evidence has emerged in recent years that several formations in the Cretaceous of Korea reveal exceptional preservation of trace fossils. This includes at least two of the lizard-track-bearing deposits: the Jinju and Haman Formations, but also include the Jindong Formation, (Fig. 1B) known for its extraordinary abundance of well-preserved tetrapod tracks^27^. Rather than declare all these deposits as Konservat-Lagerstätten in a blanket designation, we here underscore recent published assertions^24–26^ that recognize that the Jinju Formation represents the best example of a Konservat-Lagerstätten (SI) in the Sindong Group (Fig. 1B). In support of this assertion we list the ichnotaxa known from these formations (Table 1). This compilation clearly shows that the number of known ichnogenera (16) is greatest in the Jinju Formation, despite having been excavated only recently and studied for less time than other formations. More significantly the Jinju Formation has yielded tracks of twice as many major groups, including mammals, turtles, crocodylomorphs and frogs, as yet unknown in the other track-bearing formations. It has also yielded a significant number of holotypes including *N. innovatus*, ichnosp. nov., which will likely increase as the vast number of specimens already collected and presently undergoing excavation are described in detail. In addition to the diversity of tracks reported from the Jinju Formation, we note the extraordinarily high abundance^7–9^ and high quality of preservation including details of skin texture showing reticulate patterns with polygons between 0.3 and 0.5 mm in diameter^25^: see SI.

**Table 1:** Minimum diversity of named ichnogenera (some with multiple ichnospecies), and major groups represented in the four main-tetrapod track-bearing formations of Korea: Hasandong-Jingdong in ascending order

Jindong Formation	Haman Formation	Jinju Formation	Hasandong Formation
BIRDS (avian theropods) Ignotornis, Koreanaornis, Jindongornipes* Goseongornipes* Gyeongsangornipes*	BIRDS (avian theropods) Ignotornis* Koreanaornis* Jindongornipes *Goseongornipes*	BIRDS (avian theropods) *Ignotornis, Koreanaornis, Jindongornipes*	
DINOSAURS (non avian) *Brontopodus* *Caririchnium* theropod tracks	DINOSAURS (non avian) Minisauripus, Dromaeosauripus*, Grallator *Brontopodus*, *Caririchnium*	DINOSAURS (non avian) *Grallator*, *Corpulentapus*, cf*. Asianopodus*, Dromaeosauripus* Dromaeosauriformipes* Minisauripus cf. *Brontopodus*	DINOSAURS (non avian theropod tracks
	PTEROSAURS *Haenamichnus*, *Pteraichnus* LIZARDS Neosauroides koreaensis*	PTEROSAURS *Pteraichnus* LIZARDS Neosauroides innovatus* MAMMALS Koreasaltipes* TURTLES cf. *Chelichnus* CROCODYLOMORPHS *Crocodylopodus* AMPHIBIANS *Ranipes*	PTEROSAURS Pteraichnus* LIZARDS Sauripes hadongensis*
**SUMMARY**	**SUMMARY**	**SUMMARY**	**SUMMARY**
7 ichnogenera	12 ichnogenera	16 ichnogenera	2 + ichnogenera
3 holotypes	4 holotypes	4 holotypes	2 holotypes
2 major groups	4 major groups	8 major groups	~3 major groups

Compare with stratigraphy in Fig. 1B. *Indicates holotypes. Full ichnospecies names are given only for lizard tracks discussed in this paper.

### Comparison of fossil and extant lizard tracks

Although neoichnology easily correlates trackmakers with their footprints in the case of most extant taxa, including lizards^15,28^, this is rarely possible in paleoichnology at the species or genus level^15,29,30^. This is due to many factors including the incompleteness of the fossil record. For example, the maker of any given set of footprints may not be represented by body fossils, or not represented by skeletal remains including complete or even partial feet^31^. Such incompleteness applies particularly to smaller tetrapods such as lizards, with small delicate bones, which rarely survive the fossilization process, especially as articulated feet^31^. Thus, they are often under-represented in the body fossil record. The rarity of small tracks in most facies is also due to size-related bias, as the global rarity of the footprints of small Mesozoic tetrapods attests^1–9^. Even in the Cretaceous of Korea, where small tetrapod tracks are more abundant than in most other regions, they are still rare, with only three lizard^1–4^, two frog^21^ and one mammal^6^ tracksite known.

**Figure 6:**
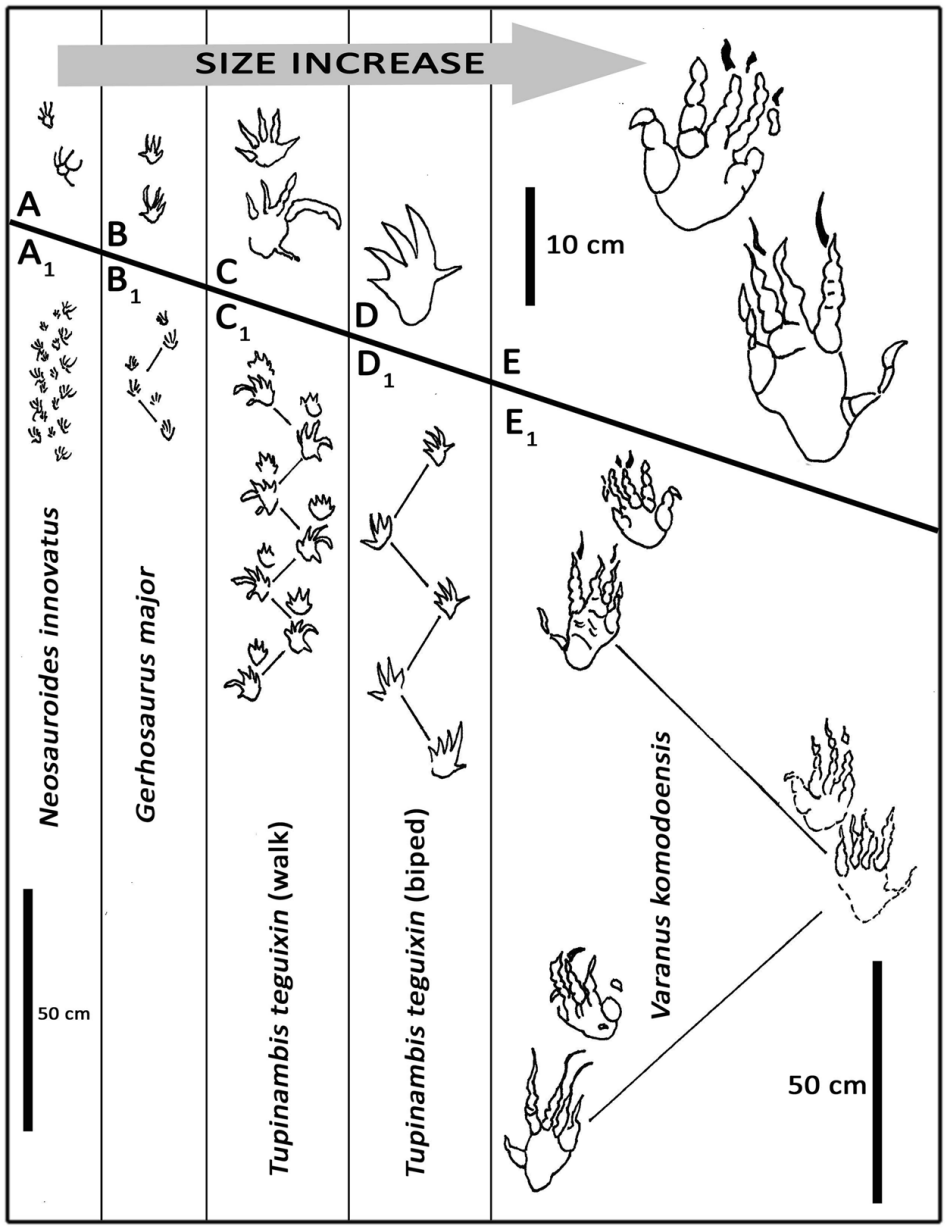
A,A_1_: the manus pes set and trackway of *Neosauroides innovatus* compared with the trackways of extant lizards (**B**–**E**) in ascending order of a size. B,B^1^: *Gerhosaurus major*^28^, C,C_1_: *Tupinambis teguixin* (walking gait)^15^, D,D_1_: *Tupinambis teguixin* so called “very quick, bipedal gait”^15^, E,E_1_: *Varanus komodoensis*. (**A**–**E**) drawn to same scale. A^1^-E^1^ drawn to same scale. Images redrawn, with permission, from various sources by M G L in Adobe photoshop (version CS6 88).

Such a sparse record makes it difficult to match fossil footprints with possible trackmakers^15^. However, the picture is by no means bleak as the tracksite database grows rapidly^1–9^. Moreover, four studies of modern lizards, considered here, have matched extant species with track morphologies^15,28,32,33^ and are therefore useful in showing the range of track and trackway morphologies (Fig. 6). These studies show that tracks of known species are not readily distinguishable from each other on morphological grounds when the trackmaker cannot be observed, or when they register tracks on different substrates^28^. For example, tracks of a meter-long Brazilian lizard *Tupinambis teguixin*, with pes and manus lengths of 9.2 and 4.4 cm respectively (Fig. 6B,C) were recorded in artificial enclosures floored with sand, soil and river mud^15^. The experiments registered several trackways from which pace angulation, stride, pace intermanus and interpes values were recorded, but concluded that it would be almost impossible to compare fossil tracks with a known species, because the same species can make quite different tracks depending on the substrate and behavior. However, while the individual tracks differ the trackway pattern (pace angulation and stride) is generally consistent and predictably variable with regard to how stride length changes to in relation speed (SI). For example, in *N. innovatus* trackway T1 (Fig. 2) the slowing down of the trackmaker in the mid section, inferred from the shorter stride, is accompanied by changes in the track registration reminiscent of the differences between the slow walking gait and very quick gait recorded for *T. teguixin*^15^. At slow gaits the wide lateral splay of the traces of pes digits IV and V is notable and similar in both *N. innovatus* and the trackway of *T. teguixin*, although in the latter the curvature of the digit IV trace is towards the postero-lateral rather than the anterolateral side. So we may infer that the lateral splay of pes digits is a function of slow speed progression^4^. Conversely the “quick”^15^ gait of *T. teguixin* (Fig. 6D) with a trackway indicating a semi bipedal gait^15^ suggests a point of comparisons with *S. hadongensis* which is interpreted as a runner^4^. For example, the two mean pace angulation values (112° and 106^o^) reported for *S. hadogensis* trackways A and B respectively^4^ can be compared with those reported for other track ways, especially the 100^o^ value for *T. teguixin* (Fig. 6D and SI Table 1). Regardless of whether trackways reveal relatively long strides and large pace angulations, or evidence of quadrupedal, semi-bipedal or bipedal gaits, there have been no numerical or absolute speed estimates for lizard progression derived from trackways. Therefore, as stressed above, all trackway-derived characterizations of speed are relative: e.g., walking or quick^15^ (SI).

As described in more detail in the supplementary information studies of modern lizard trackways have dealt with a large range of trackmaker sizes. The larger examples included a zoo-confined male Komodo Dragon (*Varanus komodoensis*), 2.54 m in length (pes length 22.0 cm) and two large Australian monitor lizards (*Varanus tristis* and *Varanus giganteus*) with pes lengths of 8.9 and 15.5 cm respectively. These trackmakers like *T. teguxin* from Brazil were clearly much larger than any of those that registered the Cretaceous trackways in Korea (pes length ~2.0- ~4.0 cm). Smaller examples of modern lizards whose tracks have been recorded and matched to known taxa include nine species with body lengths between ~25 and 50 cm and corresponding pes footprint lengths between ~2.0 and ~4.5 cm^28^. Clearly this pes size range is close to that of the Korean trackmakers, giving us a good general indication of their body lengths: i.e., ~25–50 cm.

However, turning to footprint morphology we can discount two of the nine species which were chameleons with zygodactyl feet^28^. Likewise the ground dwelling gecko *Eublepharis macularis* registered small footprints (pes length ~2.0 cm) with a posteriorly directed digit V trace, and the skink *Tiliquila scincoides* registered rounded footprints^28^. By contrast among the tracks registered by the other five lizard species (SI Table 1) the phylogenetic affinities of the trackmakers could not be discerned from their tracks, and it was concluded that the mode of locomotion and substrate has more to do with the appearance of the footprints^15,28^. The trackway of the modern lizard *Sceloporus graciosus* was compared with *N. koreaensis* to show a similarity in the mesaxony of the manus^1^. Thus, as elaborated in the supplementary information, while the tracks of extant species, can be differentiated by size and footprint morphology so as to determine general taxonomic affinity in some cases, in other cases this is not possible. Nevertheless, it is possible to differentiate trackway patterns, including step, stride trackway width and pace angulation (SI Table 1, 2) in extant and fossil trackways as a means of differentiating morphotypes, and relative speed, even if the trackmaker cannot be inferred. This is typical of the ongoing ‘Cinderella Syndrome’^34^ challenges in paleoichnology where it is rarely possible to match tracks with trackmakers at low taxonomic levels^29,30^.

In support of these inferences about the utility of lizard tracks in identifying or constraining the identification of trackmakers we have compiled morphometric information on extant lizard trackmakers from reliable sources^15,28,32,33^ (SI Table 1) for the purposes of comparison with the fossil trackways described here. The data indicate the following:

1. All four studies^15,28,32,33^ were done under controlled, not natural, track making conditions.
2. Nevertheless, all studies collected useful, if slightly different, morphometric information which facilitated comparison with extant and extinct lizard trackmakers.
3. Based on footprint size, three studies^15,32,33^ dealt with trackmakers at least twice the size of the Korean trackmakers, and in one case^32^ an order of magnitude larger.
4. Although one study^33^ dealt only with tracks in dry sand, the other three used clay-mud substrates inferred to be comparable in some respect to the pre-lithification substrates now preserved in the Jijnu Formation stratigraphic successions (Fig. 1 and SI Fig. 1).
5. Studies of extant lizard tracks and trackways show that they are sufficiently variable in size, footprint morphology and trackway configuration to differentiate morphotypes, but not to identify or discriminate between trackmakers at low taxonomic levels: e.g. species or genera.
6. The same general conclusions apply to the Cretaceous lizard tracks from Korea: i.e., they may be differentiated morphologically (and morphometrically) as the basis for ichnotaxonomy, but not used to identify trackmakers or trackmaker groups at low taxonomic levels.

## Supplementary information

Jinju Lizard Suppl Info
