# Supplementary material for: Largest Cretaceous lizard track assemblage, new morphotypes and longest trackways comprise diverse components of an exceptional Korean Konservat-Lagerstätten ichnofauna
Source: Sci Rep. 2019 Sep 16;9:13278. doi: 10.1038/s41598-019-49442-0 (PMC6746761; doi:10.1038/s41598-019-49442-0)
Supplement: Supplementary file 1 — Jinju Lizard Suppl Info [file 41598_2019_49442_MOESM1_ESM.pdf]

SUPPLEMENTARY INFORMATION

Largest Cretaceous lizard track assemblage, new morphotypes and longest trackways comprise diverse components of an exceptional Korean Konservat-Lagerstätten ichnofauna

Kyung Soo Kim[1], Jong Deock Lim[2], Martin G. Lockley[3], Dong Hee Kim[4], Laura Piñuela[5], Jae Sang Yoo[1]

[1] Department of Science Education, Chinju National University of Education, 3 Jinnyangho-ro 369beon-gil, Jinju-si, Gyeongnam, 52673, Korea

[2] Cultural Heritage Administration, Government Complex-Daejeon, 189, Cheongsa-ro, Seo-gu, Daejon, 35208, Korea

[3] Dinosaur Trackers Research Group, University of Colorado Denver, P.O. Box 173364, Denver, CO 80217-3364, USA

[4] National Science Museum, 481 Daedeok-daero, Yuseong-gu, Daejeon, 34143, Korea

[5] Museo del Jurásico de Asturias (MUJA), Rasa de San Telmo, s/n, 33328 Colunga, Asturias, Spain

INTRODUCTION

This Supplementary Information file deals with seven topics related to the main paper. All references (1-34) use the same numbering scheme as the main text.

1) Local stratigraphy of the track bearing beds which contain the lizard tracks: i.e., the type horizon of *Neosauroides innovatus* (Fig. SI 1).
2) Konservat-Lagerstätten characteristics of Jinju Formation tetrapod ichnofauna
3) Supplementary illustration of the track-bearing, type horizon in which the relationships of all trackways (T1-T5) are shown (Fig. SI 2)
4) Supplementary illustration of the holotype trackway (Fig. SI 3) with detail of selected morphometric parameters measured.
5) Supplementary illustrations and descriptions of trackways T4 and T5 (Figs S1 4-5)
6) Comparison between modern lizard trackways and three Cretaceous trackways from Korea. (Fig. 6 in main text and SI Table 1)
7) Appendix with detailed measurements of *N. innovatus* trackways T1-5

LOCAL STRATIGRAPHY

Neosauroides innovatus originates from one of four track-bearing horizons in the upper part of a 10-meter-thick stratigraphic section of alternating fine grained sandstones, shales and mudstones from which five distinct tetrapod track morphotypes were recovered (Fig. SI 1). Three *N. innovatus* trackways and two unnamed trackways (Figs. SI 3 and SI 4) originate from the third horizon in ascending order.

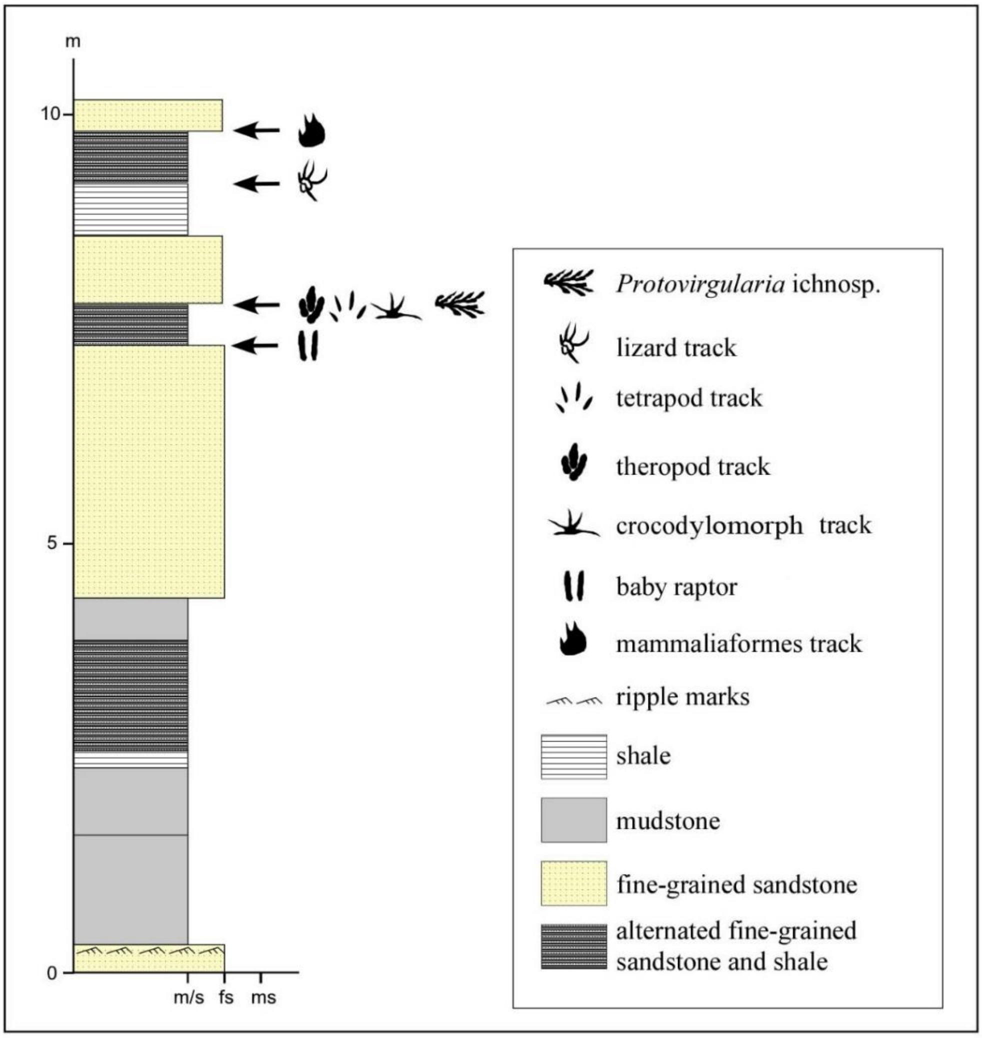

Figure SI 1. Local stratigraphy of the 2nd excavation site in Jinju Innovation City, showing the type horizon, with lizard track symbol from which the three lizard *N. innovatus* trackways and two unnamed trackways were recovered. Drawing created with Canvas X (version, 2017 Build 160, http://www.canvasgfx.com/).

KONSERVAT-LAGERSTÄTTEN CHARACTERISTICS OF JINJU FORMATION TETRAPOD ICHNOFAUNA

The term Konservat-Lagerstätten [22] has already been used several times in reference to the Jinju Formation Tetrapod ichnofauna. [5, 23- 25] As shown in Table 1 (main text) the Jinju Formation has yielded a tetrapod ichnofauna representing 8 major taxonomic groups: amphibians, turtles, lizards, pterosaurs, crocodylians non-avian dinosaurs, avian dinosaurs (birds) and mammals. This is twice the high level-taxomnomic diversity of any of the other Korean formations already well known for their diverse ichnofaunas. The Jinju Formation has yielded 16 ichnogenera, including four holotypes. The abundance of tracks representing most of these ichnotaxa is very high at multiple track-bearing levels, and has provided many specimens that form the basis of impressive museum displays and large collections at the newly-created *Jinju Pterosaur Footprint Museum*.

Diversity and abundance are not necessarily enough to designate a fossil assemblage, facies or ichnofacies as a Konservat-Lagerstätten, [22] as the quality of preservation, including soft tissue is an important factor. By definition fossil footprints register traces of soft tissue. The Jinju Formation recently yielded "exquisitely preserved" skin traces of the small theropod track *Minisauripus*,[24] the only example among 10 known assemblages, revealing perfect arrays of small (0.3-0.5 mm) integument tubercles across the entire surface of all tracks in a single trackway. Such exquisite preservation was attributed to the track-bearing surface having been a fine lacustrine mud with the consistency of a coat of wet paint less than 1.0 mm thick, and overlying a firmer layer, which we infer to have resisted indentation by small trackmakers, but not larger trackmakers[24] . This resulted in the registration of very shallow tracks from which it was difficult to obtain satisfactory 3D images using photogrammetry. This is not to say that other high resolution imaging techniques could not be more successfully applied in future.

The *Neosauroides innovatus* trackways show very similar preservation with very shallow digit traces. As with the *Minisauripus* tracks,[24] the *N. innovatus* tracks are consistently complete showing all five digit traces of manus and pes throughout most of the trackways. The majority of pes tracks in trackway T1 are categorized as having "good preservation: see section on morphometrics (below). In short, the *N. innovatus* track assemblage is both the largest known Cretaceous lizard track assemblage and the best preserved, allowing for the extraction of detailed measurements presented below in Tables SI 1 and 2. The assemblage is another pivotal data point arising from the Jinju Formation Konservat-Lagerstätten.

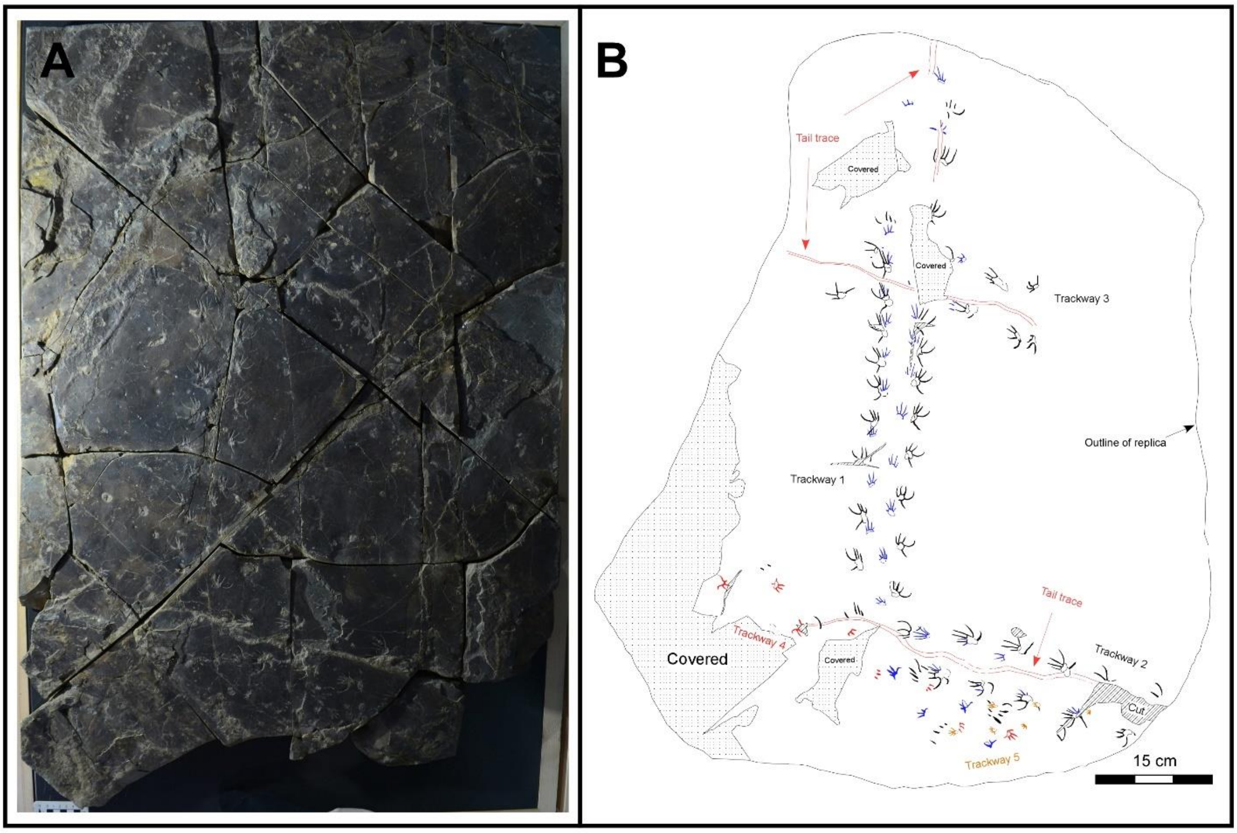

Figure SI 2. Photo and map of all five lizard trackways (T1-T5), showing covered and broken areas. Compare with Figs. 2-4 and Figs SI 3-5 for enlarged details of individual trackways. Drawing created with Canvas X (version, 2017 Build 160, http://www.canvasgfx.com/). Photograph by K-S Kim.

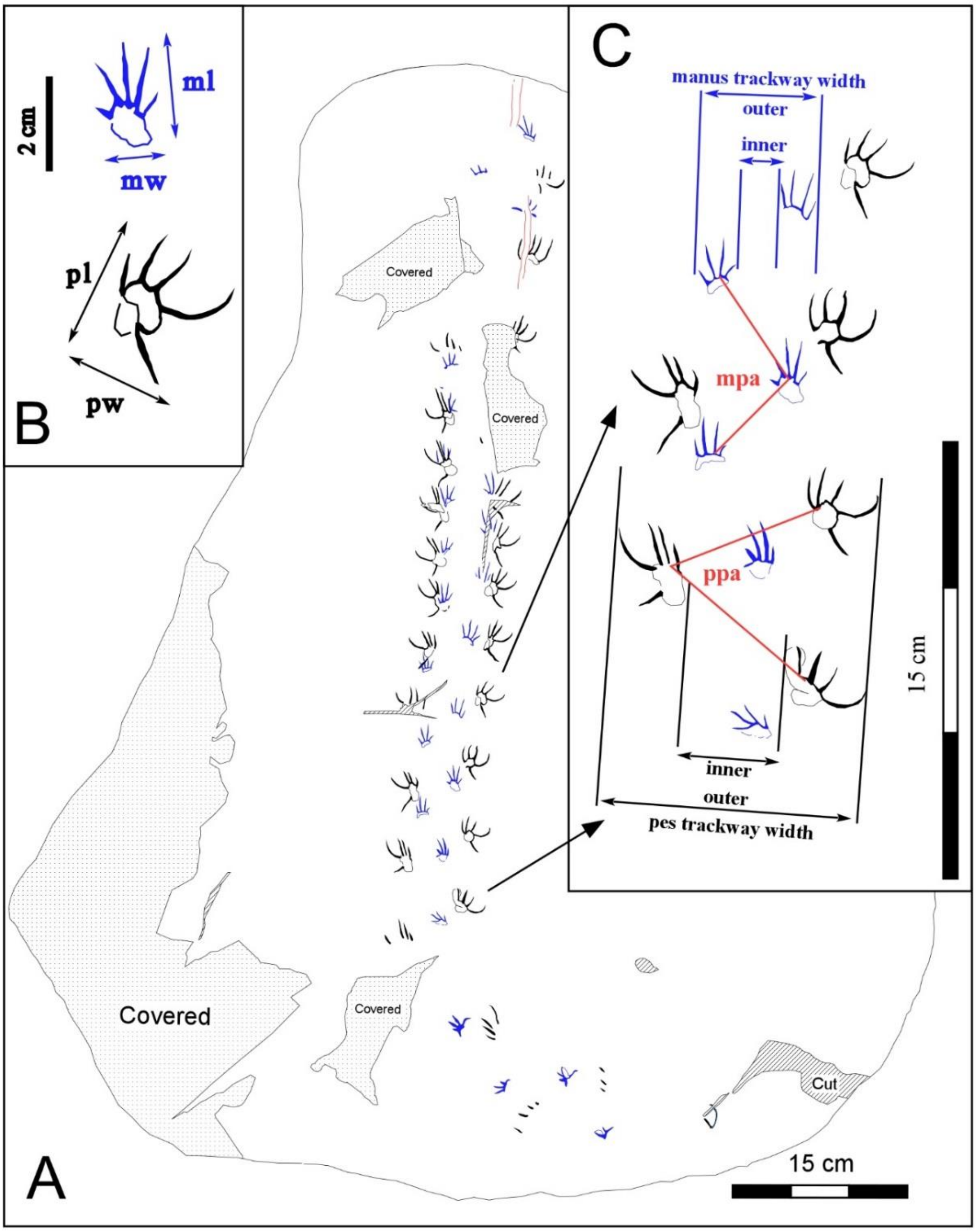

Figure SI 3. Map of trackway T1 showing pes (black) and manus (blue). B: shows details of individual manus and pes track morphology, based on well preserved tracks. C: Details of trackway parameters showing inner and outer pes and manus trackway widths, and pace angulation (PA). For clarity of methodology manus pace angulation (mpa) and pes pace angulation (ppa) are shown with red lines, indicating pace. See Table SI 1 for these and all additional morphometric measurements. Drawing created with Canvas X (version, 2017 Build 160, http://www.canvasgfx.com/).

Trackway T4

Trackway T4 (Fig. SI 4) consists of eight presumed manus tracks in a regular right-left- right sequence. The individual tracks are mostly tridactyl representing manus digits II-IV, which are aligned sub-parallel to

the trackway axis. However track number 8 (lm4) is pentadactyl and measures ~15 mm long and wide. Average step and stride 65 mm (N = 7) and 120 mm (N=6), with high pace angulation (mean 130.2°, N=7). As discussed below trackway T4 appears to represent a trackmaker smaller than those that registered trackways T1-T3).

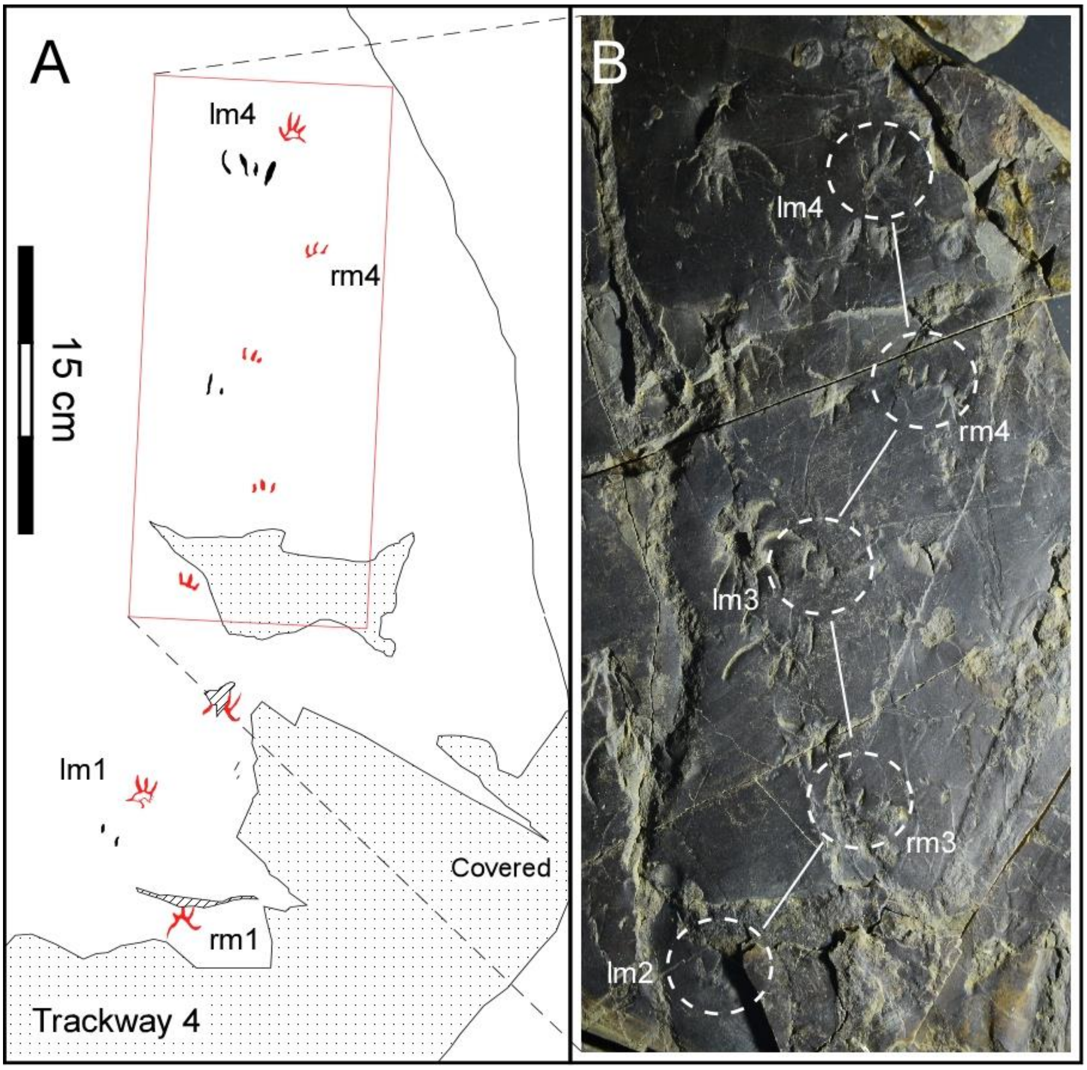

Figure SI 4. Map of trackway T4, showing regularity of inferred manus tracks, in red, and sparse representation of pes tracks, in black. See SI text for details. Drawing created with Canvas X (version, 2017 Build 160, http://www.canvasgfx.com/). Photograph by K-S Kim.

Trackway T5

Trackway T5 (Fig. SI 5) consists of six small (length ~6.0- 12.0 mm) tracks comprising a trackway slightly less complete and less regular than trackway T4. It is difficult to discern which tracks might represent manus and pes. However, there are clearly three tracks representing the right side of the trackway and three representing the left side, with an inner trackway width of ~20.0 mm. The two most distal tracks are probably manus tracks as they appear mesaxonic, elongate width digit III trace sub parallel to trackway axis. The step between these two tracks is ~6.0 cm.

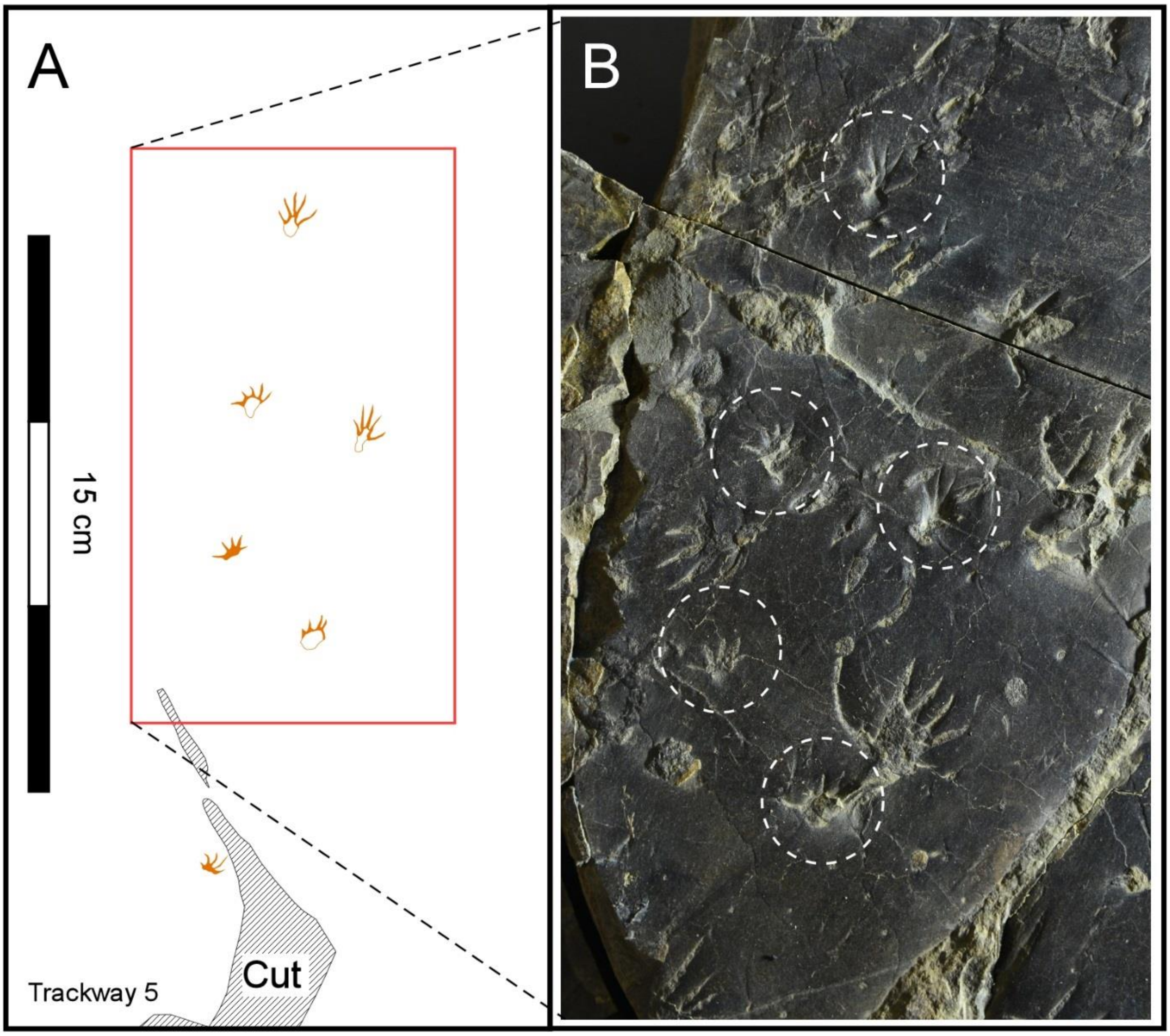

Figure SI 5. Map of trackway T5 showing incomplete and somewhat irregular trackway. See SI text for details. Drawing created with Canvas X (version, 2017 Build 160, http://www.canvasgfx.com/). Photograph by K-S Kim.

TRACKWAY MORPHOMETRICS

Available morphometric data on more than a dozen trackways of extant lizards [15,28,32-33] was compiled in Table SI 1 for comparison with the three fossil trackway morphotypes from Korea.[1,4] This database allows for detailed comparison with the large number of individual tracks in all five trackways (T1-T5), measured to show overall footprint length (FL and FW) and width, pace angulation (PA) step length (SL), individual length of pes and manus digits, divarication angles between digits I-II, II-III, III-IV and IV-V. Rotation angles of each digit trace were measured relative to the trackway mid line and inner and outer trackway widths were measured from both manus and pes. In the case of each track the quality of preservation was also noted as good, poor or very poor. For example 14 of 25 pes tracks (56%) in trackway T1 are categorized as "good" notably in the aforementioned mid-section of the trackway where the trackmaker was moving slowly: see comments on speed-related variability of extant lizard trackways in the following section. We argue that the morphometric data presented for N. innovatus (T1-T3)_ is more complete than any available for comparable lizard trackway (compare Table SI 1 and data sources with Table SI2).

COMPARING TRACKWAYS OF EXTANT AND EXTINCT LIZARDS

Two important studies of modern lizard tracks [15,28] have concluded that the morphologies of typical lizard tracks are not reliable guides to the taxonomic affinity of the trackmakers except at higher taxonomic levels: for example the tracks of chameleons, geckos and skinks made under experiential conditions can be differentiated from those of "typical" lizards. [28] Size is also a useful guide to distinguishing large varanid lizards from smaller species: [32-33] Table SI 2.

The extent to which trackways made by the same individual vary depending on behavior (speed) or substrate consistency was gauged from the study of the Brazilian lizard *T. teguixin*,[15] by studying trackways made by the same individual moving at different speeds and across different substrates. As noted in the main text, differences in speed are reflected in a predictable positive correlation between increased speed and greater stride length and higher pace angulation values. As also noted, *T. teguixin* Trackway A [15] representing a walking gait reveals widely spread pes digit traces associated with short steps and strides which resemble the tracks of *N. innovatus* in the middle part of Trackway T1. This spreading of digits appears characteristic of slow movement and contrasts with less splayed digit traces in trackway segments that represent faster progression.

The data presented in Table SI 1 indicates that all the Cretaceous lizard trackways from Korea, i.e. *N. koreaensis*, *N. innovatus* and *S. hadongensis* consistently reveal smaller track sizes and trackway parameters, such as step and stride, than almost all of the trackways of extant lizards used here for comparison. [15, 28,32-33] Given the variability of the tracks and trackways of the small extant lizards for which data is available,[1,28] i.e., *V. exanthematicus, G. major, C. zebrata, P. vitticeps* and *S. crocodilurus* and the observation that their variability to a large degree due to locomotor activity rather than taxon-specific foot morphology, it is not helpful to make detailed comparisons between the tracks of these five species and the Korean tracks. It would potentially be possible to compare digit trace lengths between all tracks, but this presupposes that the digit proportions reflect foot morphology equally, which the original study claims is not the case.[28] We nevertheless argue that it is valuable to collect all such morphometric data from tracks deemed to show good preservation which does reflect foot morphology: e.g. *N. innovatus* trackway T1 (SI Tables 1 and 2) .

Table SI 1. Comparison between published track and trackway measurements for large (varanid) and small extant lizards and the three ichnospecies from the Cretaceous of Korea (in bold). L = length, P/MTW = pes/manus trackway width. P PA = pes pace angulation. Note that six different trackways were recorded for a single individual of the Brazilian lizard *T. teguixin* including three on different substrates (sand, mud and soil). Measurements also given for three different *N. innovatus* trackways. Sources of information given in right hand column (Ref.).

Trackmaker species / **isp.**	Pes Length	Manus Length	Pes Step	Pes Stride	P/MTW	P PA	Body length	Ref.
V. komodoensis	22.0	19.0	45	89	60/40	76°	254.0	32
V. tristis	3.2-4.8	2.4-3.3	-	-	-/-		49.1-75.1	33
V. giganteus	9.4-13.0	6.0-8.5	-	-	-/-		120.4-185.0	33
V. exanthematicus	4.3	4.0	13.1	20.8	-/-	107°	48.0	28
G. major	3.8	2.8	10.6	17.6	-/-	112°	46.0	28
C. zebrata	4.2	3.4	14.5	11.5	-/-	48°	50.0	28
P. vitticeps	4.5	2.2	13.7	18.0	-/-	81°	43.0	28
S. crocodilurus	3.1	2.1	7.5	10.3	-/-	88°	34.0	28
T. *teguixin* A	9.2	4.7	18.0	23.5	68/62	88°	99.6	15
T. teguixin A ^{sand}	9.2	4.7	17.3	24.0	62/58	93°	99.6	15
T. teguixin A ^{mud}	9.2	4.7	19.6	24.1	77/58	86°	99.6	15
T. teguixin A ^{soil}	9.2	4.7	17.4	22.3	66/70	85°	99.6	15
T. teguixin B	9.2	4.7	32.9	31.0	-/-	63°	99.6	15
T. teguixin C	9.2	4.7	26.5	42.3	61/-	100°	99.6	15
N. koreaensis	**1.9**	**1.0**	**5.8**	**7.3**	**6.7/4.8**	**80°**	**-**	**1**
***N. innovatus* 1 holotype**	**2.78**	**1.72**	**5.93**	**6.48**	**9.08/4.55**	**64°**	**-**	**This study**
***N. innovatus* 2 paratype**	**4.44**	**2.01**	**8.39**	**8.94**	**11.5/7.8**	**56.7°**	**-**	**This study**
***N. innovatus* 3 paratype**	**2.39**	**1.06**	**6.48**	**6.07**	**7.5/7.2**	**49.0°**	**-**	**This study**
S. hadongensis	**2.23**	**1.92**	**4.78**	**7.92**	**106-112°/-**	**112.88°**	**-**	**2**

Table SI 2. Morphometric parameters, with means, for tracks/trackways . FL=Foot length, FW=Foot width, PL=Pace Length, PA=Pace Angle, SL=Stride length, TW=Trackway, (-) = negative inward rotation in red.

Trackway No.	Track No.	FL (mm)	FW (mm)	PL (mm)	PA (°)	SL (mm)	Length of Pes/Manus Digit					Divarica
							I	II	III	IV	V	I-II
Trackway 1	rp1	18.01	13.61				-	-	9.39	18.21	-	-
	lp1	-	-	-	-		-	-	-	-	-	-
	rp2	17.36	14.36	-	-	98.77	4.43	6.08	6.43	-	-	5.60
	lp2	10.67	29.56	65.51	98.73	-	3.24	5.35	8.36	8.03	-	3.27
	rp3	19.88	24.04	71.29	164.95	103.89	7.94	12.61	13.18	18.06	-	16.60
	lp3	20.71	23.07	110.75	80.75	180.54	6.95	13.42	15.56	9.03	-	-1.55
	rp4	20.34	30.43	55.94	73.35	114.75	9.74	11.46	9.99	17.60	-	19.57
	lp4	34.65	18.06	63.28	61.05	71.48	10.66	13.44	15.51	19.95	9.09	4.23
	rp5	28.32	25.08	55.13	62.35	60.09	11.94	13.85	9.18	18.28	11.65	25.32
	lp5	31.90	22.74	62.29	65.37	61.09	7.35	9.70	12.01	17.99	15.22	31.68
	rp6	29.58	23.16	52.63	70.28	62.19	12.46	11.94	12.20	19.09	12.05	4.00
	lp6	20.59	25.05	64.00	57.13	67.75	8.61	10.18	13.49	11.59	-	17.47
	rp7	28.84	24.20	55.48	53.43	57.63	8.42	10.21	10.91	17.90	12.52	14.38
	lp7	29.62	23.48	57.14	52.43	50.66	11.48	12.45	14.85	17.52	12.58	18.05
	rp8	32.28	20.93	51.02	49.50	47.69	10.72	43.29	12.45	19.44	14.64	13.48
	lp8	32.10	20.36	57.92	51.07	46.04	-	11.16	14.58	19.23	11.66	-
	rp9	31.25	21.55	48.26	40.47	46.63	6.96	13.04	14.54	19.90	13.56	13.73
	lp9	32.71	20.61	54.33	43.27	35.52	-	13.99	17.01	18.53	9.24	-

rp10	36.17	20.92	50.96	43.55	39.80	-	13.11	13.54	19.70	15.18	-
lp10	38.92	20.82	56.43	40.15	40.17	-	13.95	16.50	23.74	11.03	-
rp11	33.47	20.05	48.59	44.33	36.38	-	15.69	16.93	13.30	13.94	-
lp11	38.84	21.67	50.89	-	38.55	9.45	17.85	19.08	20.32	13.64	17.87
rp12	-	-	-	-	-	-	-	-	-	-	-
lp12	37.46	22.54	-	-	41.93	12.03	17.12	21.17	20.01	9.46	11.80
rp13	-	-	-	-	-	-	-	-	-	-	-
lp13	18.32	23.94	-	-	54.74	4.76	9.16	12.27	11.94	-	-4.92
rp14	31.18	20.54	54.66	-	-	10.16	16.53	12.85	19.54	7.50	1.83
lp14	-	-	-	-	-	-	-	-	-	-	-
rp15	25.07	26.55	-	-	67.99	16.58	18.37	15.95	23.39	-	22.25
lp15	-	-	-	-	-	-	-	-	-	-	-
rp16	23.84	25.37	-	-	66.52	5.12	10.57	6.11	19.30	8.15	14.55
mean	27.77	22.41	59.33	64.01	64.82	8.95	13.78	13.23	17.66	11.83	12.46
lm1	12.76	13.33	-	-		7.18	5.13	9.99	-	-	90.17
rm1	15.24	16.55	59.93	114.03		4.88	5.52	8.68	9.74	8.38	47.38
lm2	13.95	12.48	53.49	118.52	95.20	4.94	4.97	9.52	8.36	-	106.03
rm2	16.77	18.35	64.39	207.42	101.35	5.67	5.49	11.53	12.70	10.02	115.73
lm3	16.42	10.62	94.01	167.67	154.05	-	4.44	8.35	8.91	4.45	-
rm3	19.86	11.64	56.49	155.08	149.69	3.67	5.35	12.40	14.44	5.65	74.65
lm4	18.91	11.70	39.14	113.38	93.46	-	4.28	12.53	13.11	5.90	-
rm4	22.51	13.17	36.18	101.68	63.21	3.25	8.94	13.26	13.67	5.39	32.27
lm5	19.95	12.74	41.69	100.23	60.48	-	5.71	12.76	12.14	5.78	-
rm5	16.89	12.85	34.08	102.53	58.35	-	8.31	12.40	13.82	6.34	-
lm6	16.91	12.37	44.35	96.32	61.52	-	6.49	8.25	13.53	6.72	-

	rm6	20.27	16.77	43.35	97.35	65.34	-	11.14	15.86	14.28	8.61	-
	lm7	18.70	11.97	36.22	88.07	59.94	-	6.26	14.86	15.63	4.83	-
	rm7	17.80	13.23	33.59	72.50	48.81	-	2.71	9.95	18.58	5.81	-
	lm8	15.22	10.70	40.52	69.12	44.18	-	6.47	10.18	10.13	-	-
	rm8	21.42	15.96	39.62	62.87	45.47	-	5.78	13.34	13.18	11.00	-
	lm9	18.92	12.79	43.29	44.10	43.35	3.13	10.40	11.42	14.28	-	44.82
	rm9	18.79	11.83	37.26	41.52	30.76	-	5.97	16.89	19.47	-	-
	lm10	15.34	11.95	47.56	-	31.57	-	7.21	11.13	10.45	-	-
	rm10	-	-	-	-	-	-	-	-	-	-	-
	lm11	14.72	9.10	-	-	44.55	5.25	9.66	9.77	-	-	-
	rm11	-	-	-	-	-	-	-	-	-	-	-
	lm12	13.22	12.56	-	-	38.41	3.81	9.53	10.15	8.79	-	72.72
	rm12	-	-	-	-	-	-	-	-	-	-	-
	lm13	-	-	-	-	-	-	-	-	-	-	-
	rm13	13.30	22.40	-	-	-	9.76	-	5.54	6.73	5.35	-
	lm14	8.99	12.61	51.55	-	-	-	3.51	6.93	7.07	-	-
	rm14	24.10	12.72	51.54	-	65.65	-	10.57	17.63	10.94	4.31	-
	mean	17.12	13.35	47.41	103.08	67.77	5.15	6.69	11.39	12.27	6.57	72.97
Trackway 2	rp1	-	-				-	-	14.91	23.37	-	-
	lp1	35.66	30.87	84.22	59.28		7.65	12.81	14.33	21.04	6.70	1.97
	rp2	-	-	94.92	59.62	89.09	-	-	23.21	26.01	6.48	-
	lp2	47.70	28.19	82.07	52.62	88.69	12.63	19.32	15.59	21.10	21.10	9.50
	rp3	47.34	40.37	86.49	60.73	74.83	15.46	24.93	25.92	24.76	15.09	1.93

	lp3	45.91	29.44	59.62	52.60	89.09	10.38	11.94	12.01	23.48	17.17	6.73
	rp4	52.26	26.38	87.57	46.90	78.54	11.76	23.13	27.48	14.81	24.50	8.22
	lp4	43.11	25.28	89.55	59.72	70.49	-	21.54	18.00	21.40	23.56	-
	rp5	47.64	31.19	84.09	51.28	86.59	17.94	24.71	24.66	25.94	16.08	10.92
	lp5	37.66	32.05	84.24	67.92	72.85	13.11	11.75	12.57	23.49	8.87	2.50
	rp6	41.92	29.16	86.22	-	95.25	14.03	20.93	20.18	31.13	10.52	5.43
	rp7	-	-	-	-	148.11	-	-	-	24.60	-	-
	mean	44.36	30.33	83.90	56.74	89.35	12.87	19.01	18.99	23.43	15.01	5.90
	lm2	19.78	10.33				5.25	12.77	16.97	-	-	38.20
	rm3	-	-				-	-	-	-	-	-
	lm3	16.23	7.01			88.66	6.06	13.52	13.57	-	-	37.12
	rm4	18.92	12.63	76.14	60.77	-	6.34	15.17	15.41	-	-	7.45
	lm4	17.66	10.60	61.19	52.92	70.65	-	5.49	12.02	-	-	-
	rm5	16.27	11.40	80.61	62.70	65.54	-	8.19	15.36	-	-	-
	lm5	22.22	9.02	69.63	62.38	78.73	6.75	15.38	19.20	-	-	34.72
	rm6	29.82	22.03	60.89		68.02	19.12	21.00	23.05	19.27	10.21	9.12
	mean	20.13	11.86	69.69	59.69	74.32	8.70	13.07	16.51	19.27	10.21	25.32
Trackway 3	lp1	17.78	22.80				3.52	8.43	10.25	13.67	-	45.52
	rp1	16.95	19.54	66.34			7.30	9.87	9.70	11.58	-	27.30
	lp2	24.51	23.74	63.06	20.42	23.16	11.60	11.70	12.03	12.54	-	20.45
	rp2	37.37	17.53	75.18	45.83	54.98	5.46	8.28	13.02	14.84	-	21.33
	lp3	30.67	19.09	50.02	66.23	71.58	-	15.97	12.88	9.76	-	-
	rp3	-	-	63.36	63.62	60.83	-		-	21.48	-	-

	rp4	17.40	22.27	-	-	92.75	-	-	12.43	18.34	-	-
	lp5	23.03	38.69	70.89	-	-	13.99	14.72	15.60	9.46	-	72.92
	mean	23.96	23.38	64.81	49.03	60.66	8.37	11.50	12.27	13.96		37.50
	rm2	11.80	12.14				-	3.46	5.23	5.62	5.91	-
	lm3	9.32	11.89	61.14		-	-	4.90	6.43	6.39	-	-
	mean	10.56	12.02	61.14				4.18	5.83	6.01	5.91	
Trackway 4	lp1	11.59	9.65				-	-	-	-	-	-
	lp3	11.55	8.18	-	-		-	-	-	-	-	-
	lp4	13.83	28.48	-	-	114.86	11.90	6.13	11.62	13.40	-	16.65
	mean	12.32	15.44			114.86	11.90	6.13	11.62	13.40		16.65
	rm1	15.63	17.42				8.52	4.48	5.59	8.56	8.28	101.27
	lm1	13.99	15.05	68.93			-	7.42	7.14	9.25	3.18	-
	rm2	15.91	19.35	61.31	119.35	112.49	7.37	-	7.98	11.80	8.17	-
	lm2	7.72	11.39	66.99	119.03	110.61	-	3.97	5.95	6.24	-	-
	rm3	7.60	12.33	63.60	124.57	115.62	-	4.28	6.74	6.08	-	-
	lm3	6.97	10.86	68.34	137.25	122.87	-	3.40	6.18	6.13	-	-
	rm4	8.17	11.44	65.57	145.00	127.72	-	7.28	6.34	4.89	-	-
	lm4	15.59	15.49	65.66	139.43	123.09	4.20	5.33	8.33	9.17	5.80	80.67
	mean	11.45	14.17	65.77	130.77	118.73	6.70	5.17	6.78	7.77	6.36	90.97
Trackway 5	R1	6.42	6.73									
	R2	10.05	6.71									
	L2	5.98	9.78									

R3	13.49	9.23	
L3	9.32	10.96	
L4	12.25	10.38	60.60
mean	9.59	8.97	60.60
